# Why Women Wear High Heels: Evolution, Lumbar Curvature, and Attractiveness

**DOI:** 10.3389/fpsyg.2017.01875

**Published:** 2017-11-13

**Authors:** David M. G. Lewis, Eric M. Russell, Laith Al-Shawaf, Vivian Ta, Zeynep Senveli, William Ickes, David M. Buss

**Affiliations:** ^1^School of Psychology and Exercise Science, Murdoch University, Murdoch, WA, Australia; ^2^Department of Psychology, The University of Texas at Arlington, Arlington, TX, United States; ^3^Department of Psychology, The University of Colorado Colorado Springs, Colorado Springs, CO, United States; ^4^Department of Anthropology, Boston University, Boston, MA, United States; ^5^Department of Psychology, The University of Texas at Austin, Austin, TX, United States

**Keywords:** evolutionary psychology, mate preferences, lumbar curvature, high heels, physical attractiveness, cultural evolution

## Abstract

Despite the widespread use of high-heeled footwear in both developing and modernized societies, we lack an understanding of this behavioral phenomenon at both proximate and distal levels of explanation. The current manuscript advances and tests a novel, evolutionarily anchored hypothesis for why women wear high heels, and provides convergent support for this hypothesis across multiple methods. Using a recently discovered evolved mate preference, we hypothesized that high heels influence women’s attractiveness via effects on their lumbar curvature. Independent studies that employed distinct methods, eliminated multiple confounds, and ruled out alternative explanations showed that when women wear high heels, their lumbar curvature increased and they were perceived as more attractive. Closer analysis revealed an even more precise pattern aligning with human evolved psychology: high-heeled footwear increased women’s attractiveness *only* when wearing heels altered their lumbar curvature to be closer to an evolutionarily optimal angle. These findings illustrate how human evolved psychology can contribute to and intersect with aspects of cultural evolution, highlighting that the two are not independent or autonomous processes but rather are deeply intertwined.

## Introduction

“A woman’s beauty lies, not in any exaggeration of the specialized zones, nor in any general harmony that could be worked out by means of the *sectio aurea* or a similar aesthetic superstition; but in the arabesque of the spine.”

– John Updike, Pigeon Feathers and Other Stories

Women’s use of high-heeled shoes is a prevalent phenomenon in both developing and modernized societies (Miller, 1990; Freeman, 1999). In the United States alone, over $8,000,000,000 is spent annually on high-fashion footwear (Rossi, 1993). Several scholars (e.g., Roth, 1929; Smith, 1999; Morris et al., 2013; Guéguen, 2015) have advanced hypotheses about the function of high-heeled shoes for women. These ideas have ranged from the proposal of media-created associations between high heels and sexuality (e.g., Smith, 1999) to influences on specific biomechanical properties on women’s gait (e.g., Morris et al., 2013). However, these scholars have either not empirically tested their ideas or have found results suggesting that the reasons women wear high heels do *not* include the one they hypothesized (e.g., see Morris et al., 2013; Guéguen, 2015). In short, despite the widespread prevalence of high heels, the reasons *why* women wear high heels are not well understood.

Recent research by Lewis et al. (2015) may provide insight into this unexplained phenomenon. Lewis et al. (2015) took into consideration an adaptive problem uniquely faced by bipedal hominin females: (1) a forward shifting center of mass during pregnancy, and (2) a morphological adaptation that evolved to solve this adaptive problem: wedging in women’s third-to-last lumbar vertebra (Whitcome et al., 2007). These researchers reasoned that ancestral women who possessed an intermediate degree of vertebral wedging would have experienced important fitness benefits, such as being able to sustain multiple pregnancies without suffering spinal injury and being able to forage longer into pregnancy. The fitness benefits experienced by these women, in turn, would have created selective conditions for the evolution of a male mate preference for such women. Ancestrally, a woman’s lumbar curvature would have been a reliable, observable cue to her vertebral wedging (George et al., 2003). Based on this, Lewis et al. (2015) hypothesized that men have an evolved mate preference for a lumbar angle of approximately 45.5° – a value that cues the ability to shift the gravid center of mass back over the hips and simultaneously avoids the adaptive problems of excessive lumbar curvature (hyperlordosis) and insufficient lumbar curvature (hypolordosis). In support of their hypothesis, Lewis et al. (2015) found that men’s attraction toward women peaks at this angle – the optimal angle for helping ancestral women mitigate the biomechanical costs of a bipedal fetal load and minimizing the likelihood of both hyperlordosis and hypolordosis.

If lumbar curvature is an important attractiveness cue, and women possess psychological mechanisms to enhance their physical appearance (see Singh and Bronstad, 1997), then we might expect women to attempt to manipulate their lumbar curvature in ways that increase perceptions of their attractiveness. Independently, researchers interested in biomechanics and ergonomics have proposed that high-heeled shoes increase lumbar curvature (e.g., Lee et al., 2001). Together, these ideas yield a novel hypothesis about why women wear high heels: women may increase their attractiveness by manipulating their lumbar curvature with high-heeled shoes.

To test this hypothesis, we conducted two independent studies, one using archival photos from the Internet, and the second employing a controlled, laboratory-based design.

## Study 1

### Introduction

#### High Heels and Lumbar Curvature

To date, findings bearing on the relationship between lumbar curvature and high-heeled footwear have been equivocal (Russell, 2010). They have been based on small samples (e.g., 11 participants), employed measures with low validity (Fedorak et al., 2003), and produced mixed results (e.g., Snow and Williams, 1994). As such, although the belief that high-heeled shoes are associated with greater lumbar curvature is widespread, reliable evidence supporting this relationship is lacking (see Russell, 2010 for a review). The first purpose of this study was therefore to investigate the relationship between wearing high heels and lumbar curvature.

#### High Heels, Lumbar Curvature, and Attractiveness

Lewis et al.’s (2015) research establishes the importance of lumbar curvature as an attractiveness cue, but to date no studies have concurrently measured women’s lumbar curvature and attractiveness as a function of high-heeled footwear usage. The second central purpose of Study 1 was therefore to establish whether women are perceived as more attractive when they wear high heels.

### Materials and Methods

This study was carried out in accordance with the recommendations of The University of Texas at Austin Institutional Review Board. In accordance with the Declaration of Helsinki, all participants provided informed consent. Because data were collected online, participants indicated their consent electronically in lieu of providing a written signature. All participants indicated their consent in this manner, and the protocol was approved by The University of Texas Institutional Review Board.

#### Photographic Stimuli

Photographs of women wearing high-heeled and flat-soled shoes were downloaded from publically accessible websites on the Internet (links available upon request). Because individual differences in physical attractiveness are large enough to render undetectable any between-individual effects of high-heeled shoes on attractiveness (e.g., comparing the attractiveness of one woman in flats to another woman in heels), we employed a within-woman design. This required us to find images on the Internet of the same woman photographed twice, once in heels and once in flats. We also needed to be able to assess the women’s lumbar curvature, which can only be measured from the side, as in profile photographs. These constraints resulted in celebrity females representing an ideal source of data; a sufficiently wide selection of photographs of celebrity females was available on the Internet to identify two photographs of each woman, once in heels and once in flats, and both in profile. For each celebrity, we selected the first profile images that a Google search produced of the woman in heels and flats, respectively. We completed this procedure for 15 different female celebrities (list available upon request), resulting in a total stimulus set of 30 images.

#### Lumbar Curvature Measurements

We measured the women’s lumbar curvature by superimposing a virtual protractor tool (Screen Protractor, Iconico, Inc.) on a line parallel to the top of the lower back and a line parallel to the top of the buttocks, an operationalization of lumbar curvature used in clinical orthopedic settings (e.g., see George et al., 2003).

#### Raters and Attractiveness Assessments

One hundred twenty-six men (*M*_age_ = 19.77, *SD*_age_ = 4.00, range = 17–52) rated the attractiveness of the photographic stimuli. Raters were randomly assigned to either view the 15 targets wearing flat-soled shoes or to view the 15 targets wearing high-heel shoes. The images were displayed in random order to the raters, who rated the attractiveness of each target on a 10-point scale (1 = extremely unattractive, 10 = extremely attractive).

### Results

First, we set out to test whether the women’s lumbar curvature was greater while wearing high-heeled shoes than while wearing flat-soled shoes. A paired-samples *t*-test revealed that women’s lumbar curvature in high-heeled shoes (*M* = 43.37, *SD* = 9.06) was greater than their lumbar curvature in flat-soled shoes (*M* = 30.64, *SD* = 7.71), *t*(14) = 4.48, *p* = 0.001, *d* = 1.16. Second, we tested whether the women were perceived as more attractive in high heels than in flats. The women were perceived as more attractive when they were wearing high heels (*M* = 7.37, *SD* = 0.69) than when they were in flats (*M* = 6.47, *SD* = 1.11), *t*(14) = 3.10, *p* = 0.008, *d* = 0.94.

## Study 2

### Introduction

The findings from Study 1 provide the first simultaneous evidence of the relationships between (1) high heels and lumbar curvature and (2) high heels and physical attractiveness. However, these results were based on photographs that differed not only in the women’s footwear, but also in many other variables that influence women’s physical appearance (e.g., cosmetics, revealing nature of clothing). Consequently, the relationship observed in Study 1 between donning high heels and being perceived as more attractive carries with it the attendant concerns about directionality and the third variable problem. Based on these Study 1 limitations, we conducted a second, controlled, laboratory-based study to better isolate and establish (1) the effect of high heels on lumbar curvature, (2) the relationship between high heels and attractiveness, and (3) the role that lumbar curvature plays in the high heels-attractiveness relationship.

#### *Why* High Heels Increase Attractiveness

Other researchers have proposed that high-heeled shoes increase women’s attractiveness, but have either neglected to explain *why* they increase attractiveness (e.g., Roth, 1929; Smith, 1999), or have advanced hypotheses that are not consistent with extant data. For example, Morris et al. (2013) hypothesize that high heels increase women’s attractiveness through their effects on specific biomechanical properties of women’s *gait*. Consistent with the notion that high heels increase women’s attractiveness, Morris et al. (2013, p. 180) found that women were perceived as more attractive in heels. However, they found “no consistent pattern of correlations between the biomechanical measures and the judgements of attractiveness of the individual walkers.” Guéguen (2015) subsequently purported to test Morris and colleagues’ hypothesis. Guéguen conducted multiple studies documenting a link between (1) women wearing high heels and (2) men engaging in behaviors thought to be indicators of increased attraction. For example, in two studies, he demonstrated that men were more likely to be willing to participate in a survey when the solicitation to participate came from a woman wearing high heels rather than flat-soled shoes. Importantly, he obtained these results using women who were “stationed” in front of a retail store and asked passers-by to participate – he obtained these results *without gait cues*. Morris et al.’s (2013) and Guéguen’s (2015) findings that high heels increase attractiveness in the absence of gait cues provide strong evidence that the gait hypothesis, even if partially correct, cannot account for high heels’ effect on women’s attractiveness when gait cues are absent. There must be other reasons that high heels increase women’s attractiveness, which, as yet, have not been identified.

The lumbar curvature hypothesis represents one potential explanation. Moreover, the lumbar curvature hypothesis yields unique, specific *a priori* predictions about the effect of high heels on women’s attractiveness. Whereas other hypotheses generate the general prediction that women will be perceived as more attractive when they wear high heels, the lumbar curvature hypothesis offers a more nuanced set of predictions. Men do not simply prefer *greater* lumbar curvature. Rather, Lewis et al. (2015) document that men’s attraction to women increases as women’s lumbar curvature approaches a proposed theoretically optimum value—45.5°. If men are attracted to women in high heels partly because heels influence women’s lumbar curvature, then we should expect high heels to increase women’s attractiveness only when wearing heels shifts their lumbar curvature closer to the theoretical optimum, but not when the heels shift curvature away from this optimum.

To test these predictions, control for other potential high-heel-related influences on attractiveness, and rule out alternative hypotheses, we conducted a second, controlled, laboratory-based study.

### Materials and Methods

This study was carried out in accordance with the recommendations of The University of Texas at Arlington institutional review board. In accordance with the Declaration of Helsinki, all participants provided informed consent. Because data were collected online from rater participants, they indicated their consent electronically in lieu of providing a written signature. All model participants provided written informed consent. The protocol was approved by The University of Texas at Arlington institutional review board.

#### Participants

Fifty-six women (*M*_age_ = 19.36, *SD*_age_ = 1.77, age range = 18–26) were recruited from a large public university in the Southwestern United States and received partial course credit for their participation.

#### Procedure

Participants were instructed to come to their scheduled lab session in form-fitting clothing (e.g., tight jeans, yoga pants, non-baggy tee-shirts) with a pair of their own flat-soled shoes (e.g., tennis shoes). Upon arrival at the laboratory, participants were greeted by a research assistant and told that they would be participating in a study examining female appearances. Participants were taken individually to a private room to be photographed. Two photographs were taken of each participant, once in flat-soled shoes and once in heeled footwear^1^. For each photograph, the assistant instructed the participant to stand against the wall with the right side of her body facing the wall. The assistant then took a full-body profile photograph. The same instructions were used for both photographs.

#### Photographic Stimuli

**FIGURE 1:**
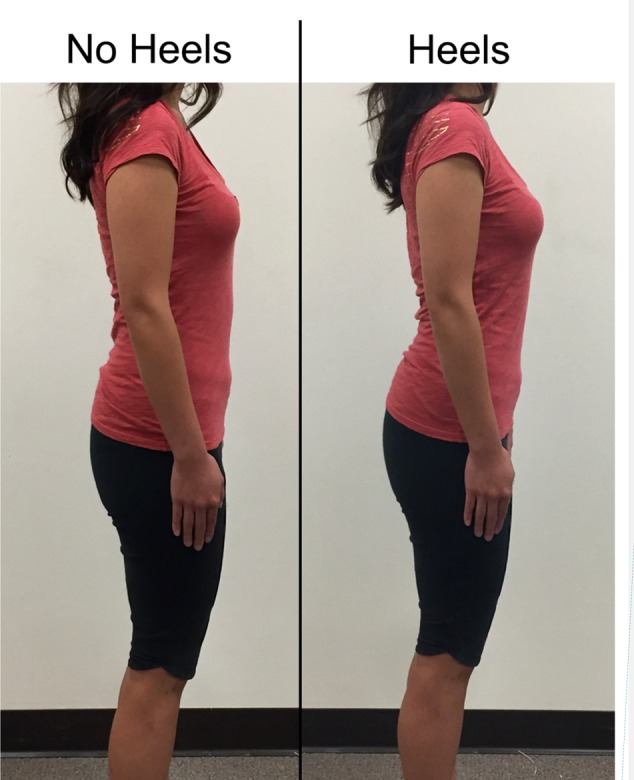
Examples of photographic stimuli. The photographs of this woman are paired here, but all 112 study photographs – one of each of the 56 women in flats and one of each woman in heels – were presented individually and in random order, with order randomized anew for each participant.

We generated a stimulus set of two images for each female participant. One image was generated from the photo of the woman in heels and the other was generated from her photo in flat-soled shoes (**Figure 1**).

To maintain participant confidentiality in the images presented to the independent sample of male raters, we deleted from each photo the portion of the woman’s body above the shoulders using the cropping tool in Adobe Photoshop. We also cropped the photos at the height of the women’s ankles. This was a critical part of study design for several reasons. First, if men exhibit a preference for women’s height, and heels modify women’s height, then any potential relationship between high-heeled shoes and women’s attractiveness could be attributable to the effect of heels on their height. Similarly, if long legs are a cue to youth (Sear et al., 2004), men have a preference for such cues in women (Swami et al., 2006; Kiire, 2015) and high heels increase the distance between the floor and the top of the leg, then any heels-attractiveness link could potentially result from these relationships. Cropping the photographs at the women’s ankles – which results in the two images of each woman having the same height and leg length – eliminates both of these potential confounds. Second, if men are attracted to high-heeled shoes themselves, independent of the effects they have on other elements of female appearance such as lumbar curvature, then the inclusion of the footwear in the photographic stimuli could influence perceptions of the women’s attractiveness.

#### Raters and Attractiveness Assessments

Eighty-two men (*M*_age_ = 20.14, *SD*_age_ = 2.43, range = 17–31) rated the attractiveness of the photographic stimuli. These participants completed the rating task online and viewed all 112 images in random order, with order randomized anew for each rater. The participants rated the attractiveness of the woman depicted in each photograph on a 10-point scale (1 = extremely unattractive, 10 = extremely attractive).

#### Lumbar Curvature Measurements

We measured women’s lumbar curvature following the same protocol as in Study 1.

### Results

#### Data Preparation

On average, women’s lumbar curvature increased in high-heeled shoes (*M* = 38.63, *SD* = 6.61) relative to flat-soled shoes (*M* = 36.45, *SD* = 6.73), paired-samples *t*(54) = 3.71, *p* < 0.001, *d* = 0.50. Within the data set, we identified three outliers who exhibited high heels-induced changes in lumbar curvature more than 2.5 standard deviations from the mean (e.g., a 10° *decrease* in lumbar curvature). We can only speculate about why high heels had such anomalous effects for these three women (e.g., they may have had very little experience wearing heels), but these aberrant data points were excluded from subsequent analyses. We computed attractiveness difference scores by subtracting each woman’s mean attractiveness rating while wearing flat-soled shoes from her mean attractiveness rating in heels. For lumbar curvature, the relevant variable was not the value of the curvature difference *per se*, but rather whether wearing high heels shifted the woman’s lumbar curvature closer to or further from the theoretical optimum of 45.5° proposed by Lewis et al. (2015). To capture this construct, we computed the absolute difference between 45.5° and the woman’s lumbar curvature in (1) flat-soled shoes and (2) high-heeled shoes, and then subtracted 2 from 1. A positive value on this index indicated that the woman’s lumbar curvature was closer to optimum in heels, whereas a negative value indicated that the woman’s lumbar curvature was further from optimum in heels.

#### Statistical Analysis

**FIGURE 2:**
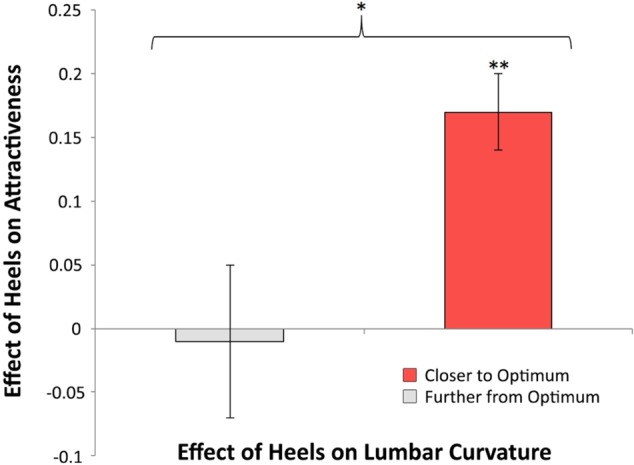
Women were perceived as more attractive in high-heeled footwear *only* when wearing heels resulted in their lumbar curvature being closer to the theoretical optimum proposed by Lewis et al. (2015). Error bars = ±1*SE*. ^∗^*p* < 0.01, ^∗∗^*p* < 0.001.

In high heels, women on average exhibited approximately 2° greater lumbar curvature (*M*_D_ = 2.41, *SE*_D_ = 0.48), *t*(51) = 5.00, *p* < 0.001, *d* = 0.69, and were perceived to be more attractive (*M*_D_ = 0.12, *SE*_D_ = 0.03), *t*(51) = 3.73, *p* < 0.001, *d* = 0.52. However, these results are not sufficient to test the prediction uniquely generated by the lumbar curvature hypothesis: that high heels’ influence on women’s attractiveness is contingent on whether wearing heels shifts the women’s lumbar curvature closer to optimum. An independent-samples *t*-test indicated that the effect of heels on women’s attractiveness differed depending on whether the women’s lumbar curvature was closer to or further from optimum in heels, *t*(50) = 2.73, *p* = 0.009, *d* = 0.84. Wearing high heels increased attractiveness *only* among those women for whom wearing heels shifted their lumbar curvature closer to optimum (*M*_D_ = 0.17, *SE*_D_ = 0.03), *t*(36) = 4.95, *p* < 0.001, *d* = 0.82; high heels were *not* associated with increased attractiveness among women whose lumbar curvature was further from optimum in heels (*M*_D_ = -0.01, *SE*_D_ = 0.06), *t*(14) = -0.17, *p* = 0.87, *d* = 0.04 (**Figure 2**).

## Discussion

The current studies provide convergent evidence across methods and independent samples of a previously untested hypothesis about why women wear high heels. These studies provide the first documented evidence of high-heeled shoes’ concurrent effects on women’s lumbar curvature and attractiveness, and reveal a precise, lumbar curvature-dependent effect of high heels on women’s attractiveness.

The current findings not only align tightly with *a priori* hypotheses generated based on evolutionary reasoning, but the design of Study 2 also intrinsically rules out several alternative hypotheses that appeal to folk psychology but have not been empirically substantiated. For example, the current findings cannot be attributed to the effect of high heels on women’s height or leg length (see Sear et al., 2004; Swami et al., 2006; Kiire, 2015). The cropping of the Study 2 photographs resulted in uniform heights and leg lengths across the within-woman photographic stimuli – yet a within-woman influence of high heels on attractiveness persisted.

Moreover, because no high-heeled shoes were present in any of the Study 2 stimuli, the current findings cannot be explained by an association between high-heeled footwear and perceptions of women’s sexuality, a media-constructed preference for high-heeled shoes, or any other reason that men might have a preference for the shoes themselves. For the same reason, hypotheses suggesting that high heels influence men’s judgments of women because of the appearance (Abbey, 1987; Abbey et al., 1987; Shotland and Craig, 1988; Koukounas and Letch, 2001; Guéguen, 2011) or color (Niesta-Kayser et al., 2010; Guéguen, 2012) of the shoes cannot account for the current findings. The current findings – which were based entirely on static images – also cannot be explained by the gait hypothesis (Morris et al., 2013; see also Guéguen, 2015). Our experimental protocol was such that it could not have been the high-heeled shoes themselves, or their influence on gait, that influenced men’s perceptions of women’s attractiveness.

The lumbar curvature hypothesis and the current studies tie together previously unrelated findings, demonstrate that several *prima facie* plausible alternative hypotheses cannot account for the observed findings, and provide a theoretically anchored explanation for one reason *why* wearing high heels affects women’s attractiveness.

### Limitations

Because we cropped the photos above the women’s ankles and did not inform the raters of the nature of the difference between the photographs, we believe that they were unaware of the type of shoe that the women were wearing and of the fact that shoe type differed across the photographs. Nonetheless, because we did not directly assess whether raters were aware of the type of shoe that the women were wearing, this is a study limitation.

Although cropping the photographs at the ankle enabled us to rule out several alternative explanations, the current research design cannot eliminate all potential confounds. For example, it is possible that in Study 2 the heels increased the women’s muscular tone. Indeed, improved muscle tone may be another reason why high heels influence women’s attractiveness. However, rather than undermining the current findings – which exhibit a lumbar-curvature dependent effect that cannot be explained by muscle tone – a consideration of heels’ effect on muscle tone may offer additional insight into the current findings. Similarly, heels may increase the protrusion of a woman’s breasts. However, like muscle tone, this cannot account for the precise, lumbar curvature-dependent effect of heels on attractiveness. Moreover, Lewis et al. (2015) demonstrated that stimuli that differed in lumbar curvature – but not breast protrusion – systematically differ in their attractiveness as a function of lumbar curvature. Future research may nonetheless benefit from attempting to disentangle these distinct potential influences of high heels on attractiveness. One possibility would be to employ photographic stimuli that present just the anterior or posterior of women’s torsos. However, such stimuli might suffer from a lack of ecological validity; presenting only half of a woman’s torso might be insufficient for activating the psychological mechanisms responsible for mate assessment. We await future research that disentangles these distinct potential influences on perceptions of attractiveness.

As predicted, heels increased attractiveness among women whose lumbar curvature was shifted closer to optimum by the shoes, but not among women whose lumbar curvature was shifted further from optimum. However, we might have expected the latter group of women to have exhibited a *decrease* in attractiveness in heels. Current findings indicate that, for these women, the shoes neither increased nor decreased their attractiveness – not what we would expect if the *only* effect of high heels on women’s attractiveness were through lumbar curvature.

Our current leading interpretation for this apparently null effect of heels for women whose lumbar curvature was pushed further from optimum is that it reflects a trade-off between the negative effects of the shift in lumbar curvature and the positive effects on other influences on attractiveness, such as muscle tone. This, of course, can only be a speculation for now and represents an important future direction. We hope that future research can further disentangle these and other potential high heel-based influences on attractiveness. For now, it is important to note that if muscle tone were the only high heels-based influence on women’s attractiveness, we should not observe the lumbar curvature-dependent effects we observed in this study. This indicates that muscle tone cannot fully account for these findings, and lumbar curvature must be at least part of the story.

### Future Directions

We hope that future research continues to investigate lumbar curvature as an important attractiveness cue—one that may provide information relevant to solving multiple distinct adaptive problems. Lewis et al. (2015) hypothesis was motivated by a consideration of the adaptive problem of a bipedal fetal load; ancestrally, a woman’s angle of lumbar curvature would have been a reliable cue to her ability to solve pregnancy-related adaptive problems. However, lumbar curvature may also communicate information about a woman’s openness to mating advances; in many other mammalian species, lordosis behavior (i.e., arching of the lower back) is a signal of sexual proceptivity (see Ågmo and Ellingsen, 2003).

Recent research (Lewis, 2017; Lewis et al., in preparation) has shown that women’s lumbar curvature increases in the presence of an attractive member of the opposite sex, an effect driven by women more strongly oriented toward short-term mating. Although Lewis and colleagues did not establish whether this shift in lumbar curvature influenced men’s perceptions of women’s attractiveness or openness to mating advances, there are theoretical reasons to believe that it might. Ancestrally, if lordosis behavior was a cue to a woman’s openness to mating advances, we should expect selection to have favored mechanisms in the male mind to attend to this type of behavior and to regulate perceptions of receptivity and attractiveness accordingly (e.g., see Goetz et al., 2012).

Future research is therefore needed to disentangle whether selection favored the evolution of male psychological adaptations to attend to women’s lumbar curvature as a cue to (1) the ability to solve pregnancy-related adaptive problems, (2) openness to mating advances, or (3) both. The possibility that lumbar curvature is a cue to both may help account for the large shift in lumbar curvature observed in the uncontrolled celebrity images (Study 1) relative to the lab-based (Study 2) images. In the lab-based study in which female participants were assigned to wear heeled footwear, it is unlikely that they were signaling interest in mating. On the other hand, the images used in Study 1 were of celebrities who had elected to dress up in high heels. The women not only would have had their lumbar curvature shifted by the shoes, but their choice to wear high heels presumably reflected their motivation to enhance their physical appearance, which could include further behavioral arching of the back. We hope to see future work disentangle the *pregnancy hypothesis* (i.e., the hypothesis advanced by Lewis et al., 2015) and the *mating interest hypothesis* – the hypothesis that lordosis behavior is a signal of mating interest in human females.

## Conclusion

The current studies illustrate how an evolutionary theoretical framework can move research toward a deeper understanding of the specific cues that influence humans’ psychology of attractiveness. By working from the starting point of a specific adaptive problem and a reliable morphological cue to the ability to solve that problem, researchers can generate tight, theoretically anchored hypotheses about specific features that should be important attractiveness cues.

We hope that evolutionary research on human standards of attractiveness will proceed in this specific, systematic manner. It is known that men prioritize physical attractiveness worldwide in mate selection (Buss, 1989), but progress hinges on identifying critical cues that make up attractiveness. Initial research in this area focused on broad categories such as cues to “health.” However, being healthy requires the organism to solve a multitude of adaptive problems, each of which may be solved by different morphological structures with distinct observable cues. By anchoring attractiveness research in cues to the ability to solve specific adaptive problems, researchers can generate more precise hypotheses and predictions (see Lewis et al., 2017, p. 364). We hope that the current studies serve as a model example of this specific and systematic approach, and make a modest contribution to our understanding of human standards of attractiveness.

## Author Contributions

DL generated the primary research hypotheses, designed the studies, and analyzed and interpreted the data. ER assisted with study design, generated study stimuli, and conducted the studies. LA-S and DB assisted with interpretation of results and manuscript preparation. ZS assisted with literature review and manuscript preparation. VT and WI assisted with conducting the study. All authors approved the final version of the paper for submission.

## Conflict of Interest Statement

The authors declare that the research was conducted in the absence of any commercial or financial relationships that could be construed as a potential conflict of interest.

## Footnotes

1 Because it was possible that not all participants owned high heels, and to control for differences in the heels they might have supplied, we provided all participants with standardized high-heeled shoes that measured 5″ from the sole to the tip of the heel. Participants were able to select the appropriate size of shoes for their feet.
